# Intelligent Badminton Training Robot in Athlete Injury Prevention Under Machine Learning

**DOI:** 10.3389/fnbot.2021.621196

**Published:** 2021-03-12

**Authors:** Jun Xie, Guohua Chen, Shuang Liu

**Affiliations:** ^1^School of Physical Education, East China University of Technology, Nanchang, China; ^2^College of Physical Education, Jinggangshan University, Ji'an, China

**Keywords:** intelligent badminton training robot, machine learning, hidden markov model, athlete injury, motion recognition

## Abstract

This study was developed to explore the role of the intelligent badminton training robot (IBTR) to prevent badminton player injuries based on the machine learning algorithm. An IBTR is designed from the perspectives of hardware and software systems, and the movements of the athletes are recognized and analyzed with the hidden Markov model (HMM) under the machine learning. After the design was completed, it was simulated with the computer to analyze its performance. The results show that after the HMM is optimized, the recognition accuracy or data pre-processing algorithm, based on the sliding window segmentation at the moment of hitting reaches 96.03%, and the recognition rate of the improved HMM to the robot can be 94.5%, showing a good recognition effect on the training set samples. In addition, the accuracy rate is basically stable when the total size of the training data is 120 sets, after the accuracy of the robot is analyzed through different data set sizes. Therefore, it was found that the designed IBTR has a high recognition rate and stable accuracy, which can provide experimental references for injury prevention in athlete training.

## Introduction

Today, people's living standards have significantly improved with the rapid development of science and technology. As one of human beings' greatest inventions in the era of artificial intelligence (AI), after years of development, robots already possess a considerable amount of AI. The robot combines high-tech technologies with multiple other technologies, including mechanics, informatics, mechanics, AI, electronics, biology, and control system engineering. Robotics has experienced several stages maturity in its research and development. At this stage, its functions are becoming more mature, and its applicational prospects are also extremely broad (Mizuno et al., 2019; Cao et al., 2020). AI has also begun to be applied in sports. There are some data analyses and intelligent sports products in the popular badminton sport, which initially demonstrate the appeal of applying AI technology to traditional sports (Mutaqin et al., 2020). At present however, the application of AI in badminton sports is limited in both depth and breadth. Most badminton training methods are still realized with the one-to-one or one-to-many manual teaching methods, which can be extremely restrictive to the improvement of a players' own level (Chia et al., 2019; Gao et al., 2020). Therefore, applying machine learning and other related algorithms to the training of badminton players has become the focus of scientific research in recent years.

Badminton is a net-to-net confrontation sport, and the athletes on both sides compete in skill and tactics without physical contact. Therefore, the possibility of injury due to collision is very small in badminton. However, athletes are required to play in a short time, combining the consistency of running and jumping, and the upper limb variability of lower limbs to complete the game (Huang et al., 2019; Wang et al., 2019; Mansec et al., 2020). Therefore, improper operation during training may cause physical injury to the athlete. Preventing physical injury of the athletes is therefore particularly important. Machine learning is an important algorithm in AI. Many scientific researchers have adopted machine learning to study the action and behavior recognition of the human body. For example, standing, walking, running, and lying down in daily actions can be recognized and classified by algorithms (Li and Zhang, 2017; Polydoros and Nalpantidis, 2017; Wang et al., 2018). Similarly, machine learning can also detect abnormalities and falling in the human body, mainly by collecting human electromyographic signals, acceleration signals, and video signals. For badminton, table tennis, tennis, football, and other complex activities, the wearable acceleration sensor can be adopted to collect the data, and then the machine learning and other related algorithms are adopted for recognition and classification (Li et al., 2019b). Machine learning and AI technology therefore have broad applicational prospects in the field of sports.

In summary, the recognition and regulation of actions during sports is of extreme significance, so as to reduce the damage caused to badminton players during training and to further improve their abilities. The innovation of this study lies in the design of the IBTR system, and the use of HMM in the machine learning algorithm to recognize movements of athletes. Finally, computer simulation is adopted to test and analyze its performance in multiple directions, which provides experimental evidence for the future development of sports and injury prevention in athletes during training.

## Literature Review

### Research Trends in the Application of Machine Learning

As the core content, machine learning has been applied to various branches of AI, and there are many studies based on it. In wireless networks, Jiang et al. (2017) adopted machine learning techniques to propose applying them in attractive applications in 5G networks, including cognitive radio, massive multi input multi output (MIMO), femto /Small cells, heterogeneous networks, smart grids, energy harvesting, and communication between devices, to meet the diverse needs of wireless networks, which provided experimental support for extremely high speeds and new applications of future wireless networks. In the medical field, Walsh et al. (2018) used machine learning to predict the suicidal behavior of adolescents through the collection of clinical data, and screened the risks of non-fatal autotomy attempts of the adolescents through a retrospective analysis of historical data. In electric power, Dalal et al. (2019) proposed a data-driven distributed opportunity-constrained optimization equation and built an agent that predicts the results of the operation process for the power system using machine learning, so as to solve the traction in large-scale power grids; the simulation results revealed that the proposed solution is cheaper and more reliable than other candidate solutions. In the field of physics, Namba et al. (2020) took the three-dimensional alignment control of asymmetric top molecule sulfur dioxide (SO_2_) as an example to study a machine learning method for drawing landscape maps in a low-dimensional control parameter space, and studied the control of a set of mutually orthogonal linearly polarized laser pulses; these pulses were parameterized by time delay and fluence ratio; the parameters could be represented by points in the parameter space or time and frequency resolved spectra; and the simulation analysis disclosed that the machine learning model based on convolutional neural network (CNN) is trained using a small number of training samples, and can construct mapping with sufficient accuracy to predict temperature-related control mechanisms.

Based on the analysis of scholars in the above-mentioned related fields, machine learning is an important algorithm in AI. Although its fields of application involve wireless communications, medical and health, power grids and physics, the training and movement analysis in sports basically rely on the traditional coaching experience and training and skill improvement. Therefore, it is of great significance to apply AI technologies, such as machine learning, to sports such as badminton like we have done in this study.

### Research Status of Badminton Robot

As a product of multiple disciplines, the badminton robot involves a variety of theoretical knowledge. Generally speaking, the robot is large in size, and requires engineers from multiple disciplines to collaborate and discuss to complete all the work. The badminton robot developed by Sapiee and Annuar (2018) is composed of a robotic arm fixed on the rails of the court and an off-court processor. During training, the shuttlecocks flight trajectory is read through the two off-court cameras; the data is processed in the off-court desktop computer and is then transmitted back to the controller to control the motor-driven manipulator to hit the shuttlecock, which can realize the function of playing against an opponent (Sapiee and Annuar, 2018). Mizuno et al. (2019) developed a fully automatic badminton game robot, which could receive and serve in the same environment as the human athlete; the Kinect sensor and the space shuttle in this system were applied to predict and detect the motion trajectory of the shuttlecock. Liu et al. (2020) collected depth images of the scene using a depth camera and applied the machine vision theory to process the obtained depth images. The position of the badminton camera coordinate system in the three-dimensional space was obtained by combining the image depth information. The position of the field coordinate system was thus realized. Finally, the position information of the shuttlecock in the multi-frame image was adopted to predict the drop point of the shuttlecock; then, the badminton robot quickly ran to the predetermined position to complete the hitting task (Liu et al., 2020).

In summary, the above-mentioned research shows that related machine learning has been applied to many fields, and the development and performance of badminton robots are constantly improving. However, there are relatively few related studies about applying the machine learning to predict the shuttlecock trajectory. Therefore, the machine learning is adopted to recognize the movement of badminton players and to predict the badminton trajectory to realize the hitting task and sparring effect with the players, which has important value for the application of AI in sports.

## Method

### Design Idea of Intelligent Badminton Training Robot

In the era of intelligence, the development and promotion of intelligent robots has become an important direction for the transformation and upgrade of the domestic sports industry. For example, various ball pitching machines, ball pickers, and human-robot sports robots have gradually entered professional sports and ordinary sports practices. Badminton is a national sport that is suitable for all ages and can involve several people synchronously (Sato et al., 2017). With the rapid development of science and technology, automatic machines used for training, fitness, and entertainment (such as badminton ball machines and badminton companion robots) have been successfully developed and have been applied to a certain extent. However, there are not many types of badminton pitching machines, and the pitching system is not perfect. It is not only because the service life of the badminton robot is reduced due to the serious damage caused by the pitching equipment, but also because its intelligence is low.

Therefore, a new type of badminton training robot was researched, developed, and designed from both hardware and software aspects, and was applied in this study in the training of badminton players, enhancing the function and performance of the pitching system.

### Hardware Design of the Intelligent Badminton Training Robot

**Figure 1:**
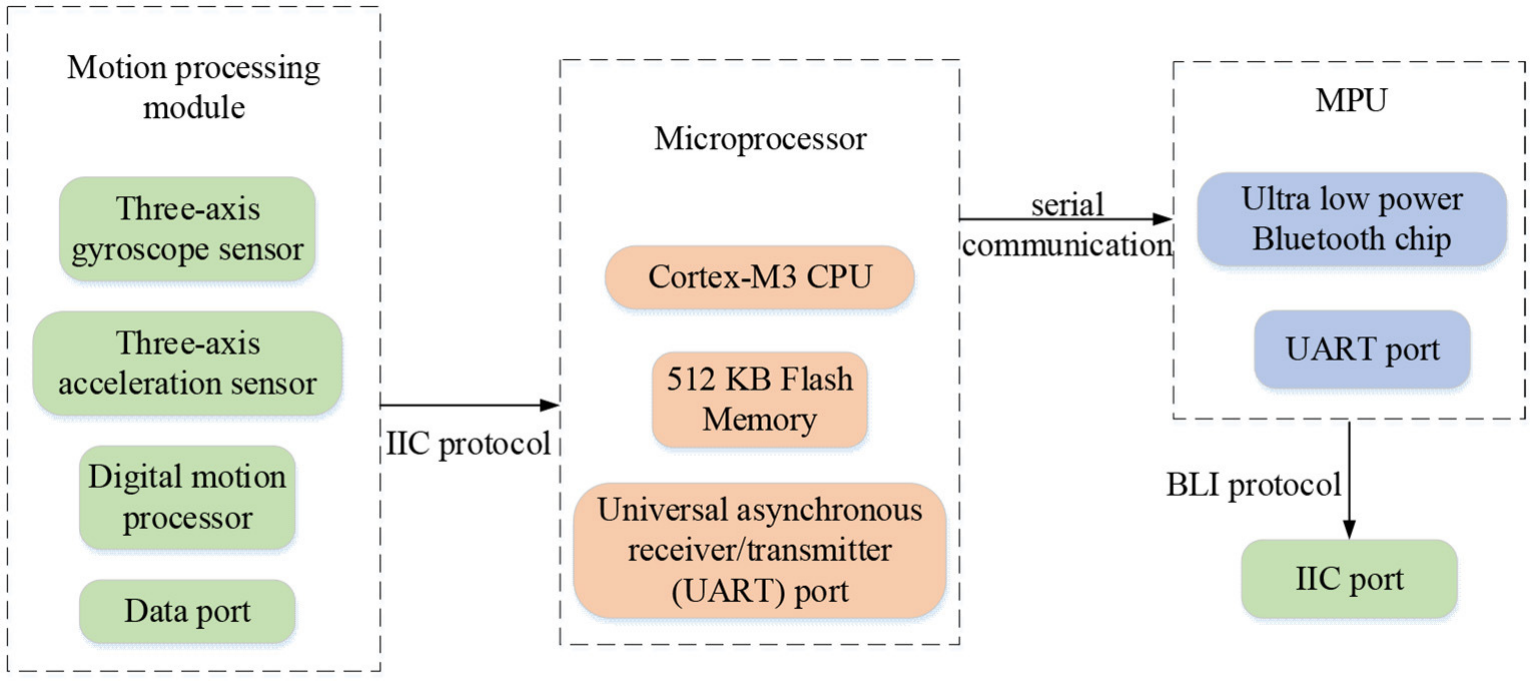
The hardware framework of the intelligent badminton training robot.

In the designed badminton robot, the hardware mainly includes two aspects: the swing movement of the badminton racket and the movement of the mobile chassis for the robot. During the movement of the badminton racket, the module fixed on the bottom of the racket collects the three-axis acceleration and the three-axis angular acceleration in real time and receives the original data through the integrated digital motion processor (DMP) (Lau et al., 2018) of the module for next posture fusion. Then, the calculated motion posture data is transmitted to the main control chip through mobile communication, and then the main control chip recognizes the motion mode through the machine learning algorithm. Finally, the final result is sent to the upper computer through the Bluetooth module for the user to observe. The hardware framework of the IBTR is shown in Figure 1.

The mobile chassis model of badminton requires the speed of each wheel axis and the roller speed to calculate the true speed of the wheel (Matsuo, 2017; Singh and Singh, 2018). First, a plane coordinate system is established to decompose the movement of the chassis into three independent components: translation along the X axis, translation along the Y axis, and rotation along the yaw axis. *v*_*t*_*x*__, from left to right, refers to the movement speed of the chassis on the X axis, and *v*_*t*_*y*__ from the bottom to the top, refers to the movement speed of the chassis on the Y axis. $\vec{\omega }$ takes clockwise rotation as the positive direction, and refers to the rotational angular velocity of the yaw axis. The speed expression can be written as:

(1) $$\begin{array}{l} \vec{v}={\vec{v}}_{t}+\vec{\omega }\times \vec{r} \end{array}$$

In the above equation, $\vec{v}$ represents the vector where the center of the chassis points to one of the wheel axis; $\vec{r}$ represents the motion speed vector of the wheel axis; and ${\vec{v}}_{t}$ refers to the speed vector at time *t*. The following equation can be obtained by decomposing $\vec{v}$ along the X and Y axes:

(2) $$\begin{array}{l} \left\{ \begin{array}{c} {\vec{v}}_{x}={\vec{v}}_{t_{x}}+\omega \times r_{y}\\ {\vec{v}}_{y}={\vec{v}}_{t_{y}}+\omega \times r_{x} \end{array}\right. \end{array}$$

According to the above-mentioned wheel axis speed, the speed ${\vec{v}}_{⊥}$ perpendicular to the direction of the roller and the speed ${\vec{v}}_{\|}$ of the roller movement direction can be decomposed. Since ${\vec{v}}_{⊥}$ is provided by the friction between the ground and the roller during the movement, its value can be adapted to the needs as long as the maximum friction is large enough. The value of ${\vec{v}}_{\|}$ can be derived according to the following equation:

(3) $$\begin{array}{l} {\vec{v}}_{\|}=\vec{v}\cdot \overset{⌢}{u}=-\frac{1}{\sqrt{2}}v_{x}+\frac{1}{\sqrt{2}}v_{y} \end{array}$$

Where, $\overset{⌢}{u}$ is the unit vector along the direction of the roller, so the speed of the wheel can be obtained as below:

(4) $$\begin{array}{l} v_{\omega }=\frac{{\vec{v}}_{\|}}{\text{cos }45{}^{\circ}}=\sqrt{2}\left(-\frac{1}{\sqrt{2}}v_{x}+\frac{1}{\sqrt{2}}v_{y}\right)=-v_{x}+v_{y} \end{array}$$

In this study, a = 320 mm and b = 290 mm can be obtained based on the installation position of the wheels on the chassis of the vehicle. Based on the above-mentioned wheel axis speed and roller speed, the rotating speeds of four wheels can be expressed as Equation (5) below:

(5) $$\begin{array}{l} \left\{ \begin{array}{c} v_{\omega_{1}}=v_{t_{y}}-v_{t_{x}}+\omega \left(a+b\right)\\ v_{\omega_{2}}=v_{t_{y}}+v_{t_{x}}\text{-}\omega \left(a+b\right)\\ v_{\omega_{3}}=v_{t_{y}}-v_{t_{x}}+\omega \left(a\text{-}b\right)\\ v_{\omega_{4}}=v_{t_{y}}+v_{t_{x}}\text{-}\omega \left(a\text{-}b\right) \end{array}\right. \end{array}$$

In the cylindrical coordinate system, the coordinate (x, y, z) is to represent the point *P*, *P*_*z*_ refers to the position of the point *P* on the *Z* axis, and *d* represents the projection length of the vector *P* on the plane XOY. The angle between X axis and the vector *P* on the XOY axis is expressed with α (Victor et al., 2018). Then, the cylindrical coordinates of point *P* can be expressed with *z*, α, *d*, as follows:

(6) $$\begin{array}{l} \left\{ \begin{array}{c} 0\leq d\leq +\infty \\ 0\leq \alpha \leq 2\pi \\ -\infty <z<+\infty \end{array}\right. \end{array}$$

The position can be represented by the cylindrical coordinate, which can also be regarded as the x-axis translation for a certain length, rotation around the z-axis, and translation along the z-axis on the Cartesian coordinate system (Chen et al., 2019). In order to facilitate the association of the positions in the Cartesian coordinate system and the cylindrical coordinate system, point *P* can be expressed as the following equation:

(7) $$\begin{array}{l} Cyl\left(p_{z},\alpha ,d\right)=Trans\left(0,0,p_{z}\right)Rot\left(A,\alpha \right)Trans\left(d,0,0\right) \end{array}$$

In the above Equation (7), *Cyl*(*p*_*z*_, α, *d*) refers to the position in cylindrical coordinates, and *Trans*(0, 0, *p*_*z*_) refers to its translation transformation. Of which, the translation along the X, Y, and Z axes can be represented by *P*_*x*_, *P*_*y*_, and *P*_*z*_, respectively, and the rotation transformation is represented by *Rot*(*A*, α). The rotation axis is the A axis, and α refers to the rotation angle.

When the robot receives and sends instructions for routine operations, the robot's posture changes according to the instant instructions due to the different description instructions for the robot. This is related to the steel body of the robot (the object with fixed dimensions, fixed size, and a fixed form, and its basic properties and volume are not easy to change under the action of force) (Zagatto et al., 2018). By connecting many steel bodies, the movement posture and position of the object can be described using the Cartesian coordinate system, so as to complete the corresponding command actions, which mainly include translational coordinate transformation, rotation coordinate transformation, composite coordinate transformation, and homogeneous coordinate transformation (Kawata et al., 2019).

### Software Design of the Intelligent Badminton Training Robot

**Figure 2:**
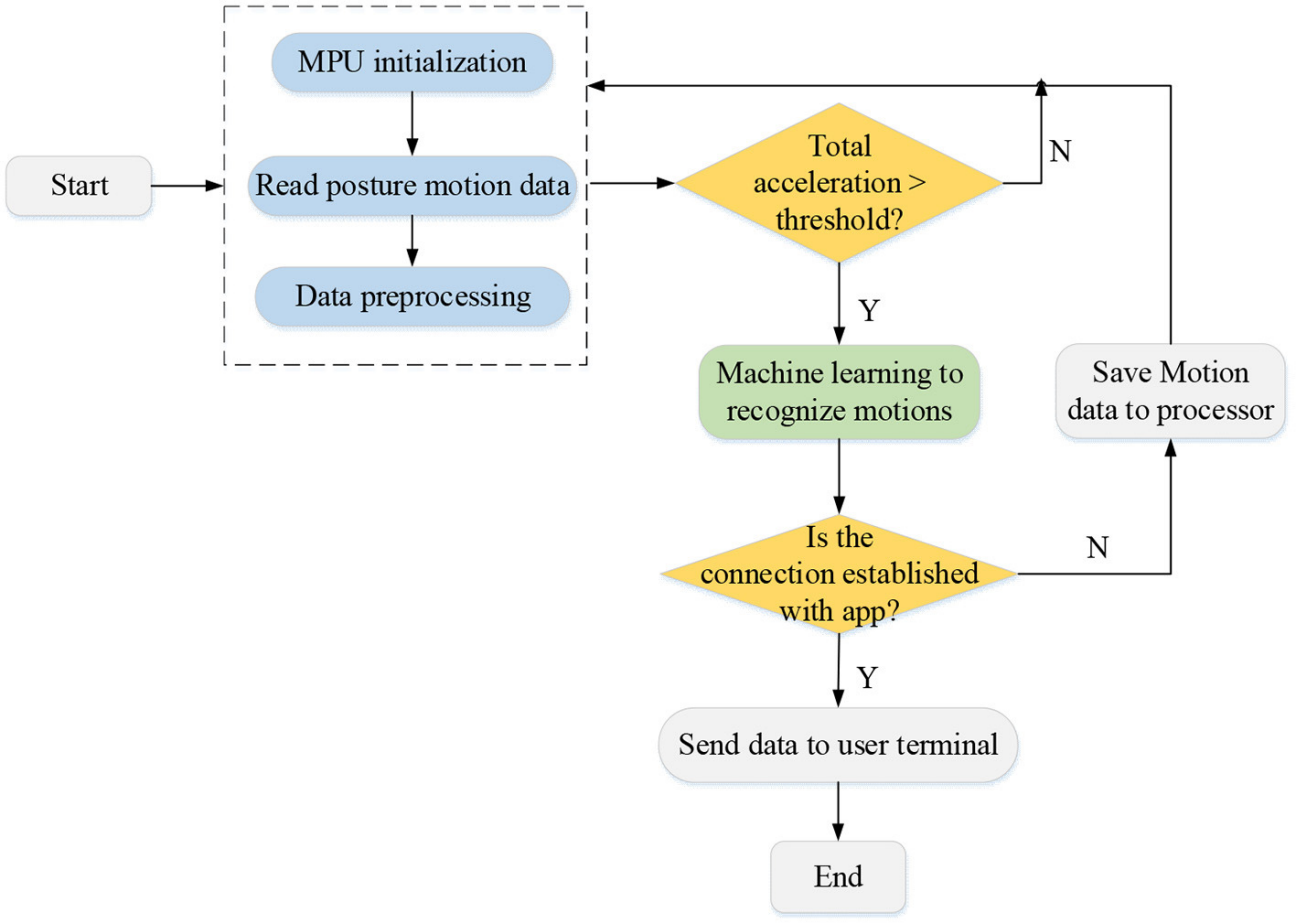
The flowchart of the software control system of the intelligent badminton training robot.

In the designed badminton robot system, its software system mainly includes four parts: the machine supervised learning system, the posture calculation system, the Bluetooth communication system, and the Android user terminal software. The software design of the control system is mainly developed around the machine learning system. The control chip receives the three-axis posture angle and three-axis acceleration transmitted by the movement processing module through the Inter-Integrated Circuit (IIC) communication protocol and processes the sensor data through machine learning algorithms to analyze the physical fitness and swing movements of the player. The flowchart of the software control system is shown in Figure 2.

#### Data Collection and Pre-processing

**Figure 3:**
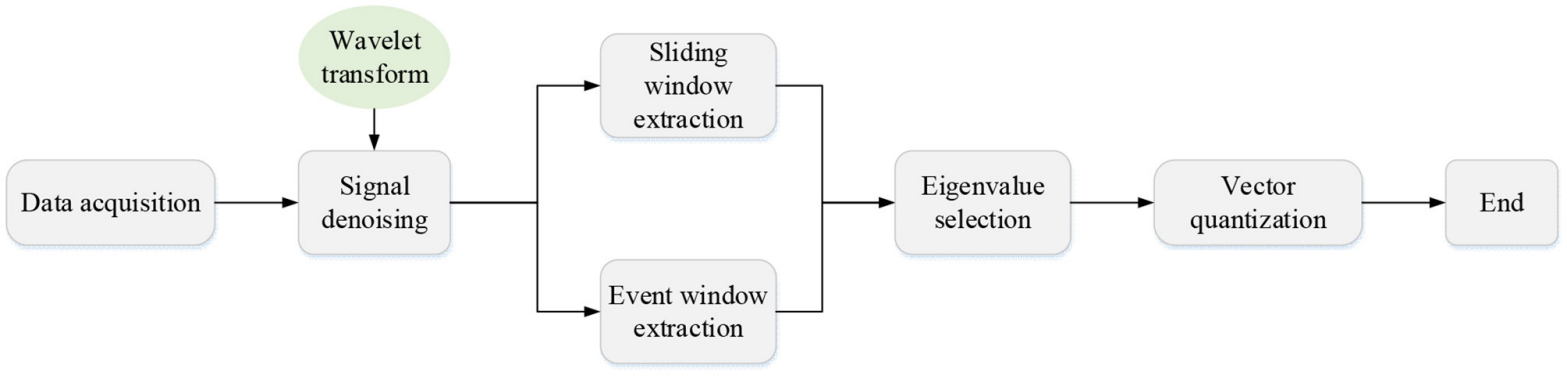
The data pre-processing process.

After the controller of the intelligent robot is started, the Microprocessor Unit (MPU) data processing module is first initialized, and then the current three-axis acceleration, three-axis angular acceleration, and geomagnetic declination are measured by its own inertial elements and Hall sensors. The internal integrated digital motion processing is adopted for posture fusion, and the three-axis posture angle is calculated. Finally, the obtained data is transmitted to the main control chip through the serial port, thereby completing the data collection of the intelligent feather robot. After the data collection is completed, the signal denoising algorithm (Li et al., 2019a) is adopted to denoise the original data and the data information of a single shot action is extracted with the window segmentation technology (Liang et al., 2019). Next, the single hitting motion is processed by time series and framing to obtain multiple meta-motions. The accuracy of k-means clustering (Mao et al., 2019) is evaluated on multiple waveform features (such as peak values and average values) of each meta-action. The feature with the most obvious clustering characteristics is selected as the characteristic information of each meta-motion. The meta-action is assigned to the corresponding codebook space with vector quantization, and the observation sequence of the hitting motion can be obtained as the input of the subsequent algorithm model. The data pre-processing process is shown in Figure 3.

#### Motion Recognition Based on Machine Learning

Due to the time sequence of the shuttlecock motion, a probability model about time sequence (named as HMM) is adopted to model the hitting motion. The recognition process of the racket motion is to extract the observation sequence of the current hitting motion through the pre-processing algorithm as the input of each established hitting motion HMM. In addition, the Viterbi algorithm (Vu et al., 2018) is utilized to obtain the appearance probability of the best state sequence for the current hitting motion under each model. The hitting motion of the model with the largest probability output result is deemed as the recognition result of the current observation sequence.

HMM modeling mainly relies on three parameters: initial probability, transition probability, and observation probability. A set of applicable HMM is established for each hitting motion, and each hitting action is divided into n meta-motions with sequence. The codebook corresponding to each meta-motion is determined by vector quantization, which is considered to be the observation set defined for HMM. Therefore, each hitting motion can be regarded as a set of observation sequences with the length of n. For this observation sequence, the parameters of each model are trained through the Baum-Welch algorithm (Jalal and Kim, 2020) until the parameters can meet the convergence requirements.

The Baum-Welch algorithm is an unsupervised model learning and training algorithm. It is assumed that a given training data value contains *S* observation sequences of length *T* {*O*_1_, *O*_2_, ..., *O*_*S*_} without a corresponding state sequence, then the parameters of the model can be learned with the forward algorithm and the backward algorithm. The observation sequence data is regarded as observation data *O*, and the state sequence data is regarded as unobservable hidden data *I*, so the following equation can be obtained:

(8) $$\begin{array}{l} P\left(O|\lambda \right)=\underset{I}{\sum }P\left(O|I,\lambda \right)P\left(I|\lambda \right) \end{array}$$

The parameter learning can be realized by the expectation-maximization algorithm (EM algorithm) (Polydoros and Nalpantidis, 2017), in which, E finds the Q function $Q\left(\lambda ,\bar{\lambda }\right)$, as follows:

(9) $$\begin{array}{l} Q\left(\lambda ,\bar{\lambda }\right)=\underset{I}{\sum }\log P\left(O,I|\lambda \right)P\left(O,I|\bar{\lambda }\right) \end{array}$$

In the Equation (9), $\bar{\lambda }$ refers to the current estimated value of the model parameter, and λ refers to the maximized model parameter. The M in the EM algorithm is to obtain the model parameter λ = (*A, B*, π) by maximizing the Q function $Q\left(\lambda ,\bar{\lambda }\right)$. First, the parameter π_*i*_ is calculated using Lagrangian multiplication to find the partial derivative of it, then the following equation can be obtained:

(10) $$\begin{array}{l} \pi_{i}=\frac{P\left(O,\;i_{1}\;=\;i|\bar{\lambda }\right)}{P\left(O|\bar{\lambda }\right)} \end{array}$$

The parameter A can then be obtained by the Lagrangian multiplication of the constraint condition $\overset{N}{\underset{j=1}{\sum }}a_{ij}=1$. The *a*_*ij*_ can be expressed as Equation (11):

(11) $$\begin{array}{l} a_{ij}=\frac{\overset{T-1}{\underset{t=1}{\sum }}P\left(O,\;i_{t}\;=\;i,\;i_{t\;+\;1}\;=\;j|\bar{\lambda }\right)}{\overset{T-1}{\underset{t=1}{\sum }}P\left(O,\;i_{t}\;=\;i|\bar{\lambda }\right)} \end{array}$$

Finally, the parameter B is obtained. The constraint condition is set to $\overset{M}{\underset{k=1}{\sum }}b_{j}\left(k\right)=1$, and the partial derivative of *b*_*j*_(*o*_*t*_) to *b*_*j*_(*k*) is not 0 when *o*_*t*_ = *v*_*k*_ is satisfied, which is represented by *I*(*o*_*t*_ = *v*_*k*_). The following equation can be obtained:

(12) $$\begin{array}{l} \begin{array}{c} b_{j}\left(k\right)=\frac{\overset{T}{\underset{t\;=\;1}{\sum }}P\left(O,\;i_{t}\;=\;j|\bar{\lambda }\right)I\left(o_{t}\;=\;v_{k}\right)}{\overset{T}{\underset{t=1}{\sum }}P\left(O,\;i_{t}\;=\;j|\bar{\lambda }\right)} \end{array} \end{array}$$

However, there are multiple data sets for the same hitting motion in the sample in the designed IBTR, but the training HMM parameter model is based on a single sample, so the model data may fall into the local optimum, and the recognition rate of other samples is low. Therefore, two schemes are proposed for multi-sample training. One scheme refers to the mean training method. The input of the model (the observation sequence of the sample) is treated to realize the data layer fusion, and the model training is realized after the average of multiple sets of training data is obtained under the same model. The other scheme refers to the frequency weighted training method. The frequency of the sample observation sequence in the model is adopted for linear weight, and the parameters in the Baum-Welch algorithm are corrected. It is supposed that the sequence of M observation values *O*^(*m*)^ (*m* = *1, 2, … m*), and *P*_*m*_ is the frequency of the *mth* observation sequence, then the correction equations are given as follows:

(13) $$\begin{array}{l} {\bar{\pi }}_{i}=\overset{M}{\underset{m}{\sum }}\frac{\alpha_{1}^{\left(m\right)}\left(i\right)\beta_{1}^{\left(m\right)}\left(i\right)}{P\left(O^{\left(m\right)}|\lambda \right)} \end{array}$$

(14) $$\begin{array}{l} {\bar{a}}_{ij}=\frac{\overset{M}{\underset{m}{\sum }}P_{m}\overset{T_{m}-1}{\underset{t=1}{\sum }}\alpha_{t}^{\left(m\right)}\left(i\right)a_{ij}b_{i}\left(O_{t+1}^{\left(m\right)}\right)\beta_{t+1}^{\left(m\right)}\left(j\right)}{\overset{M}{\underset{m}{\sum }}P_{m}\overset{T_{m}}{\underset{t=1}{\sum }}\alpha_{t}^{\left(m\right)}\left(i\right)\beta_{t}^{\left(m\right)}\left(j\right)} \end{array}$$

(15) $$\begin{array}{l} {\bar{b}}_{j}\left(k\right)=\frac{\overset{M}{\underset{m}{\sum }}P_{m}\overset{T_{m}-1}{\underset{t=1}{\sum }}\alpha_{t}^{\left(m\right)}\left(i\right)\beta_{t}^{\left(m\right)}\left(j\right)I\left(o_{t}\;=\;v_{k}\right)}{\overset{M}{\underset{m}{\sum }}P_{m}\overset{T_{m}-1}{\underset{t=1}{\sum }}\alpha_{t}^{\left(m\right)}\left(i\right)\beta_{t}^{\left(m\right)}\left(j\right)} \end{array}$$

In the above equations, $\alpha_{t}^{\left(m\right)}\left(i\right)$ and $\beta_{t}^{\left(m\right)}\left(j\right)$ are the forward probability and backward probability of the *mth* sequence, respectively. ${\bar{\pi }}_{i}$, *ā*_*ij*_, and ${\bar{b}}_{j}\left(k\right)$ are the revised initial, transition, and observation probability parameters, respectively.

#### Bluetooth Communication Connection

After the control system is started, its working mode is automatically set to allow surrounding devices to initiate connections. After the connection is successful, the control system transmits motion analysis data to the integrated memory through the serial port and sends it to the mobile phone application (APP) through the low-power Bluetooth protocol. If it fails to connect to the APP, the microprocessor will save the data in the integrated Flash memory (Steels et al., 2020) inside the processor. When the control system establishes a connection with the mobile phone APP, it will immediately send the unsent data to the client APP through the Bluetooth function. In addition, the control system will clear the data that has been uploaded to the client APP to avoid duplicate data recording.

### Running Simulation of Badminton Robot

The performance of the IBTR was simulated and analyzed in this study. Multiple sets of controlled experiments were designed to test the motion recognition accuracy of the system under different training data sizes. Six representative basic motions were selected based on the rules and playing methods of badminton, including high and far ball, smash, flat driving, flat block, lifting, and chop. These motions were demonstrated by a professional badminton player and 30 records were collected as the training sample set of the system. In the simulation, the accuracy on the different training set sizes (N = 120 and 300 groups, respectively) and recognition rate on different dimensions of the system were mainly determined and analyzed comprehensively. Of which, the recognition rate of the IBTR was compared from three dimensions, as shown in Table 1.

**Table 1:** Multiple-dimensional comparison of recognition rate of the intelligent badminton training robot.

**Dimension**	**Method**
Data pre-processing	Sliding window segmentation based on hitting time
	Sliding window segmentation based on the maximal value
	Window segmentation based on the event
Motion recognition	Motion recognition based on support vector machines (SVM)
	Action recognition based on traditional HMM
	Action recognition based on improved HMM
Recognition of training objects	Same athlete
	Different athletes

## Results and Discussion

### Comparison of Recognition Rates in Different Dimensions

The recognition rate of IBTR is compared and analyzed from the three dimensions: data pre-processing, motion recognition, and recognition of training objects.

**Figure 4:**
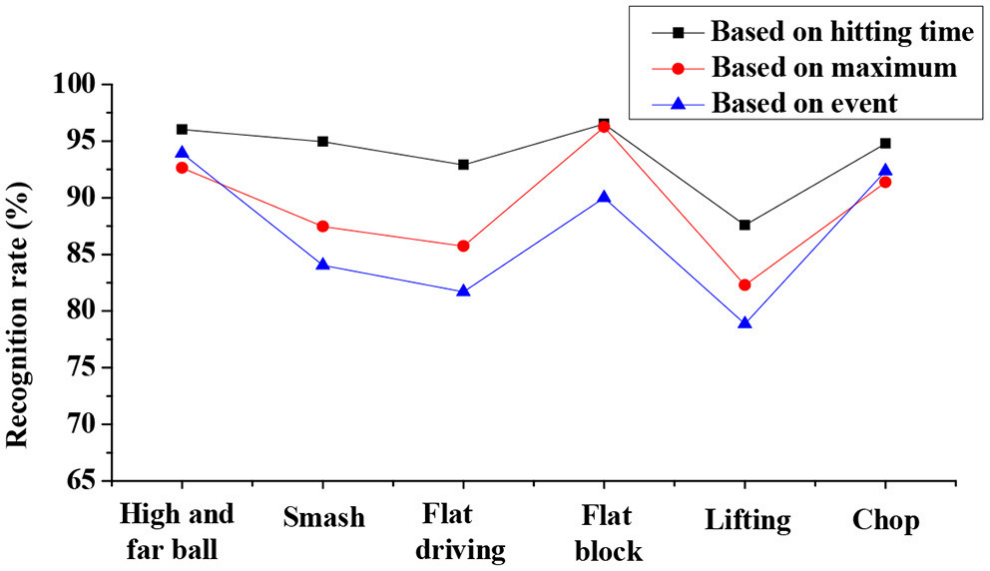
Analysis on recognition rate of the intelligent badminton training robot in the data pre-processing dimension.

**Figure 5:**
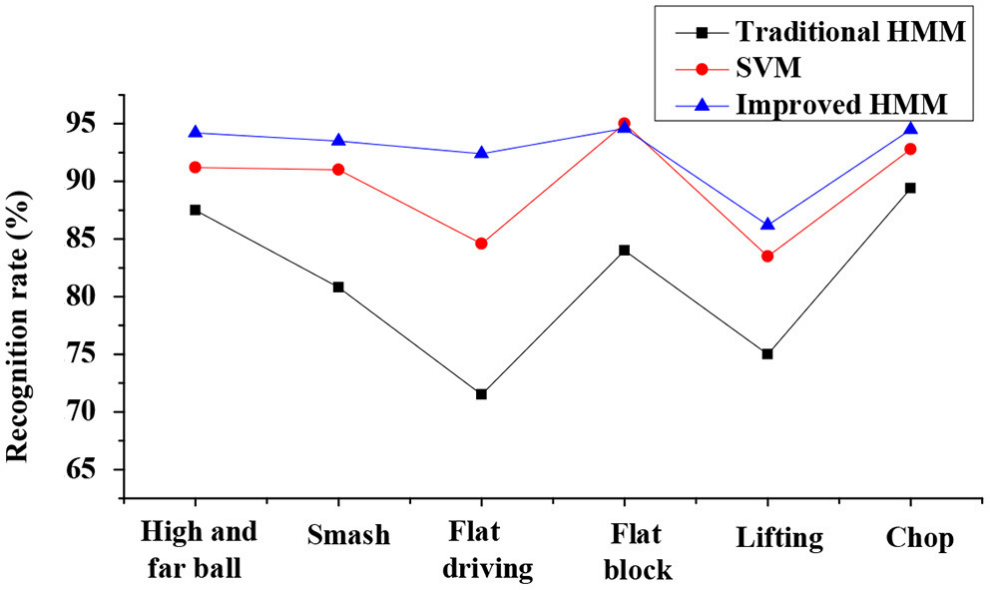
Comparison on recognition rates of the intelligent badminton training robot with the three training algorithms.

**Figure 6:**
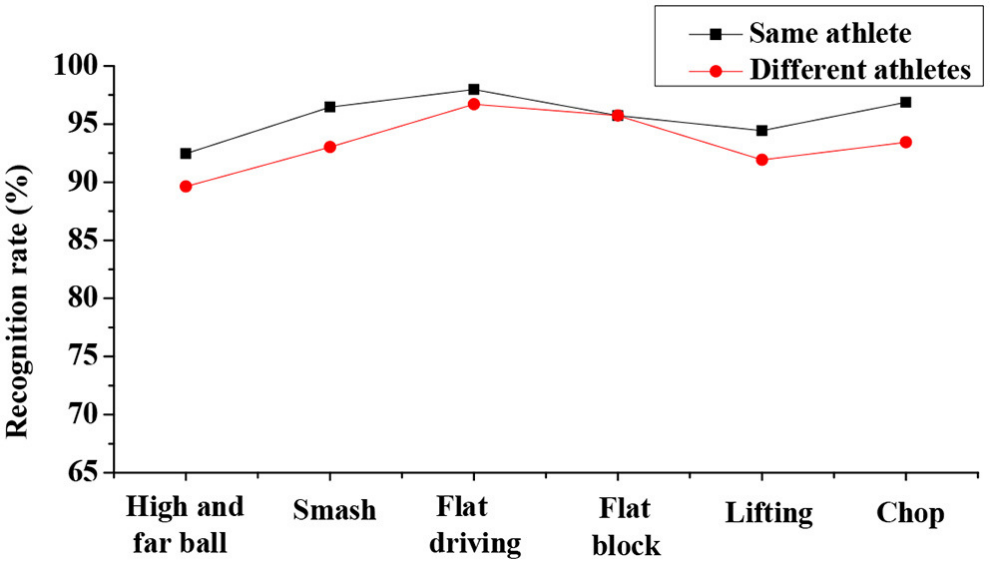
Analysis on recognition rates when the object is a same athlete or different athletes.

Analysis on recognition rate of the IBTR in the data pre-processing dimension discloses that the sliding window segmentation based on the hitting motion has a higher recognition rate than the other two dimensions, and the highest recognition rate can reach 96.03% (as shown in Figure 4). The recognition rate in the motion recognition dimension is compared and analyzed based on the SVM, traditional HMM, and improved HMM. The results indicate that the traditional HMM training has the lowest recognition rate, followed by SVM, and improved HMM action has the highest recognition rate, which can be up to 94.50% (as given in Figure 5). In terms of the recognition of training objects, the recognition rate based on the same athlete is up to 97.98%, and the average recognition rate of the system based on different athletes is up to 96.711%. Thus, it is obvious that recognition rate is highest when the subject is the same athlete (as illustrated in Figure 6 below).

### Comparative Analysis of Model Simulation Accuracy

**Figure 7:**
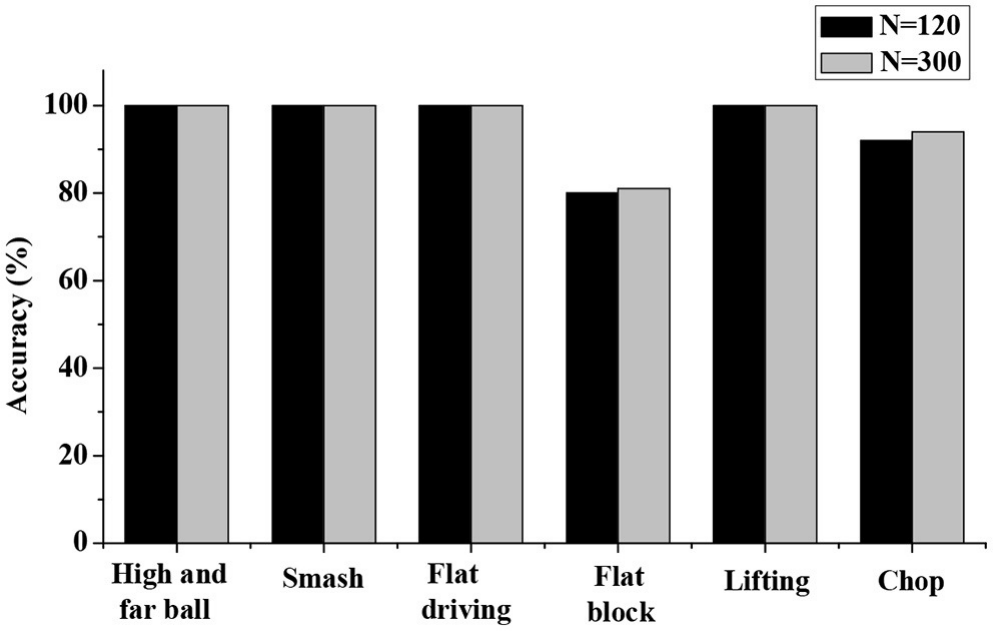
Comparative analysis on accuracy of the model in different data set sizes.

In this study, the training data set size N is selected as 120 groups and 300 groups for the simulation test, and the results are shown in Figure 7. The simulation test results indicate that the test accuracy for most types of motions reached a higher level when the size of the training data is 120 sets. In addition, the improvement in accuracy is extremely insignificant when the size of the training data is increased to 300. Therefore, the system takes into account the operation efficiency and stability. When the total scale of the training data is 120 groups, that is, the training data scale of each action type is 20 groups, the accuracy rate is basically stable.

### Discussion

Analysis on recognition accuracy of the IBTR reveals that the recognition accuracy of the data pre-processing algorithm, based on the sliding window segmentation, at the moment of hitting can reach 96.03% after the optimization of the HMM model, and the application of the improved HMM algorithm shows a recognition rate for the robot of 94.50%. Thus, the recognition effect to the training set samples is good. In addition, there is no obvious difference between the recognition accuracy in whether the recognition object is the same athlete or not, and the recognition of each action is above 90%. This obviously shows that in the IBTR designed in this study, its recognition rate is significantly improved, and the recognition effect of the training set samples is good after the parameters of each dimension are adjusted and optimized.

Therefore, by applying the designed IBTR to the daily training of badminton players it can better recognize the athletes' movements, and it can significantly reduce the athletes' injuries during the training process.

## Conclusion

The overall hardware and software framework of the IBTR system was first designed in this study, and the HMM under the machine learning algorithm was adopted to recognize the motions of the athletes. The designed IBTR was then tested and analyzed multi-directionally in terms of motion recognition accuracy, using the computer simulation. Finally, it was found that the recognition rate and accuracy of the designed IBTR are both high, and the system runs stably, which provides an experimental reference for the injury prevention in athlete training. However, there were also some shortcomings in this study. For example, the study focuses on the design of the IBTR and is less concerned with the cooperative training of athletes. In addition, the specific calculation method of the three-axis posture angle is relatively complicated in the hardware design of the IBTR, and is therefore not clearly pointed out in this study. Therefore, a cloud platform for big data analysis will be built in follow-up research for data mining and data analyses on a large amount of player games and training data, so as to analyze the technical advantages and shortcomings of various athletes, thereby promoting the ability of athletes faster and preventing the injury of athletes.

## Data Availability Statement

The raw data supporting the conclusions of this article will be made available by the authors, without undue reservation.

## Author Contributions

All authors listed have made a substantial, direct and intellectual contribution to the work, and approved it for publication.

## Conflict of Interest

The authors declare that the research was conducted in the absence of any commercial or financial relationships that could be construed as a potential conflict of interest.
